# Atheroprotective Effect of Fucoidan in THP-1 Macrophages by Potential Upregulation of ABCA1

**DOI:** 10.3390/biomedicines11112929

**Published:** 2023-10-30

**Authors:** Zeenat Mirza, Dalal A. Al-Saedi, Salma Saddeek, Sanaa Almowallad, Rehab F. AlMassabi, Etimad Huwait

**Affiliations:** 1 King Fahd Medical Research Center, King Abdulaziz University, Jeddah 21589, Saudi Arabia; zmirza1@kau.edu.sa; 2 Department of Medical Laboratory Sciences, Faculty of Applied Medical Sciences, King Abdulaziz University, Jeddah 21589, Saudi Arabia; 3 Department of Biochemistry, Faculty of Sciences, King Abdulaziz University, Jeddah 21589, Saudi Arabia; 4 Cell Culture Lab, Experimental Biochemistry Unit, King Fahd Medical Research Centre, King Abdulaziz University, Jeddah 21589, Saudi Arabia; 5 Department of Chemistry, Faculty of Sciences, University of Hafr Al Batin, Hafr Al Batin 39511, Saudi Arabia; salmayms@uhb.edu.sa; 6 Department of Biochemistry, Faculty of Sciences, University of Tabuk, Tabuk 48322, Saudi Arabiarf-saif@ut.edu.sa (R.F.A.)

**Keywords:** fucoidan, Ox-LDL, SR-AI, LXR-α, CD36, ApoA1, THP-1 macrophages, foam cells

## Abstract

Targeting foam cells reduces the risk and pathophysiology of atherosclerosis, of which they are one of its early hallmarks. The precise mechanism of action of fucoidan, a potential anti-atherogenic drug, is still unknown. Our objective was to assess the ability of fucoidan to regulate expression of ATP-binding cassette transporter A1 (ABCA1) in ox-LDL-induced THP-1 macrophages. Molecular docking was used to predict how fucoidan interacts with anti-foam cell markers, and further in vitro experiments were performed to evaluate the protective effect of fucoidan on modulating uptake and efflux of lipids. THP-1 macrophages were protected by 50 µg/mL of fucoidan and were then induced to form foam cells with 25 µg/mL of ox-LDL. Expression levels were assessed using RT-qPCR, and an Oil Red O stain was used to observe lipid accumulation in THP-1 macrophages. In addition, ABCA1 protein was examined by Western blot, and cellular cholesterol efflux was determined using fluorescently labeled cholesterol. Under a light microscope, decreased lipid accumulation in ox-LDL-induced-THP-1 macrophages pre-treated with fucoidan showed a significant effect, although it did not affect the expression of scavenger receptors (*SR-AI* and *CD36*). It is interesting to note that fucoidan dramatically increased the gene and protein expression of *ABCA1*, perhaps via the liver X receptor-α (*LXR-α*). Moreover, fucoidan’s ability to increase and control the efflux of cholesterol from ox-LDL-induced THP-1 macrophages revealed how it may alter ABCA1’s conformation and have a major effect on how it interacts with apolipoprotein A (ApoA1). In vitro results support a rationale for predicting fucoidan and its interaction with its receptor targets’ predicted data, hence validating its anti-atherogenic properties and suggesting that fucoidan could be promising as an atheroprotective.

## 1. Introduction

Atherosclerosis is a complicated disease initiated by the deposition of lipids into the subendothelial blood vessels, which causes oxidization of low-density lipoprotein (ox-LDL) and leads to inflamed lesions. Subsequently, monocytes migrate into intima and differentiate into macrophages. Upon macrophages’ uptake of ox-LDL lipids, their transformation into foam cells results in an accumulation of foam cells that start going through apoptosis and necrosis [1].

Macrophage foam cells are a hallmark of atherosclerosis in its early stages, which results from a pathological imbalance in the increased uptake of ox-LDL or decreased cholesterol efflux. Consequently, to maintain homeostasis, cholesterol uptake and efflux are carefully balanced [2]. Lipids enter the cytoplasm of macrophages through scavenger receptor (SR)-dependent phagocytosis and macropinocytosis processes. Macropinocytosis is membrane ruffling, a component of fluid-phase nonspecific endocytosis that is dependent on phosphoinositide 3-kinase (PI3K) [3,4]. SR class AI (SR-AI) and the cluster of differentiation 36 (CD36) are transmembrane glycoproteins and master receptors for cholesterol uptake in macrophages and are responsible for 70–90% of ox-LDL uptake; other SRs cannot compensate for their absence [5]. Atherosclerosis may be successfully treated by increasing cholesterol efflux through ATP-binding cassette transporter A1 (ABCA1)-mediated active transfer from macrophages to extracellular space. The released cholesterol can then be easily accepted by apolipoprotein A (ApoA1) or high-density lipoprotein (HDL) cholesterol [6]. The ApoA1 interacts with ABCA1 to generate nascent HDL, which is subsequently used by ATP-binding cassette sub-family G member 1 (ABCG1) to promote cholesterol efflux, hence implying that ABCA1 and ABCG1 work sequentially, and ABCA1 is a significant transporter mediating cholesterol homeostasis by reverse cholesterol transport [7,8].

Statins are lipid-lowering medications that block the activity of 3-hydroxy-3-methyl-glutaryl-coenzyme A (HMG-CoA) reductase and are frequently used as first-line therapy [9]. Although statins have shown some efficacy in reducing LDL, they have some drawbacks, mainly muscular damage and liver toxicity [10]. Hence, there is an urgent need for safer alternative therapeutics. Fucoidan is a bioactive natural marine compound. These sulfated polysaccharides in brown macroalgae have shown promise in reducing atherosclerosis [11,12,13]. Nevertheless, it is crucial to understand the molecular mechanisms that underlie its beneficial effects.

Due to its varying molecular weight, monomeric composition, sulfate position, and degree of sulfation, fucoidan’s structure-activity has anti-atherogenic properties in ox-LDL-induced THP-1 macrophages. In this study, we have chosen fucoidan from *Fucus vesiculosus* to evaluate how it affects cholesterol homeostasis in THP-1 macrophages (particularly ABCA1 regulation in THP-1 macrophages) through gene expression analysis and computational prediction to enhance our understanding of its mechanism. Since macrophages play a crucial role in the formation of foam cells and the pathophysiology of atherosclerosis, they were used for the in vitro experiments performed to determine the validity of understanding the molecular mechanism of fucoidan. To our knowledge, no prior research has examined how fucoidan, when administered as a protective agent, affects ABCA1 regulation in THP-1 macrophages.

## 2. Methods

### 2.1. Identification of Atherosclerosis-Related Proteins

Genes encoding proteins implicated in atherosclerosis in humans and their models (THP-1 cell) were retrieved from GeneCards V5.11.0 [14]. Common genes were selected to integrate with each atherosclerosis listing stage by Venny 2.1 [15] for choosing candidate targets.

### 2.2. Protein—Ligand Docking

Molecular docking is a computational method for prescreening molecules in the drug discovery process by determining the binding affinity of the protein–ligand complex, which aids in the selection and filtration of potential inhibitors against the therapeutic biomarkers. Fucoidan and palmitic acid (CID: 129532628 and 985, respectively) structures were retrieved from NCBI’s PubChem database. The 3D x-ray structures of SR-AI, CD36, and the cryo-EM structure of ABCA1 were obtained from RCSB’s Protein Data Bank [PDB ID: 7DPX (2.0 Å), 5LGD (2.07 Å), and 5XJY (4.10 Å), respectively] [16,17,18]. Protein structures were prepared by selecting chain A, adding hydrogen atoms, and removing crystallographic waters, heteroatoms, and unwanted co-crystallized ligands if present. Docking was performed with a monomeric unit of fucoidan to calculate the binding energy (kcal/mol) from the ligand–protein interaction using AutoDock 4.2.6 [19]. Parameters were set as protein-rigid and ligand-flexible, and grid box dimensions were created according to the binding pocket of each receptor. The binding free energy of the protein–ligand complex was used to score various configurations. The optimum position was selected based on the lowest docking energy and RMSD of less than 2 Å [20]. The docking results were then analyzed using PyMOL (version 2.5.2, Schrödinger, LLC) and LigPlot+ (version 2.2.8, European Bioinformatics Institute). Additionally, loss in solvent-accessible surface area (SASA) was calculated using Discovery Studio Visualizer V21.1.0 as: ΔSASA = SASA of protein—SASA of a protein–ligand complex.

### 2.3. Protein—Protein Docking

Protein–protein docking was utilized to create an interaction model between ABCA1–ApoA1. Binding sites of ABCA1 and ApoA1 (PDB ID: 5XJY (4.10 Å) and 3R2P (2.20 Å)) [18,21] were identified and then docked using ClusPro 2.0 servers [22,23]. The best-docked complexes were chosen based on the largest member size and lowest energy from ClusPro. Moreover, protein–protein docking was performed using the HDOCK server to validate [24]. The docked complexes were visualized and the interacting amino acid residues were analyzed using the PDBsum database and PyMOL [25].

### 2.4. Monocyte Culture and Treatment

A human THP-1 monocytic cell line was provided by the Cell Culture Lab, Experimental Biochemistry Unit, King Fahd Medical Research Centre (KFMRC), King Abdulaziz University, Jeddah, KSA. THP-1 was maintained in 1X RPMI Medium 1640 supplemented with 10% fetal bovine serum (FBS), 100 U/mL of penicillin–streptomycin (100 U/mL), and 200 mM L-glutamine (GibcoTM, Thermo Fisher Scientific, Waltham, MA, USA). All the materials used to supplement the medium were purchased as sterilized. Cells were incubated at 37 °C in a humidified atmosphere containing 5% CO_2_.

**Figure 1:**
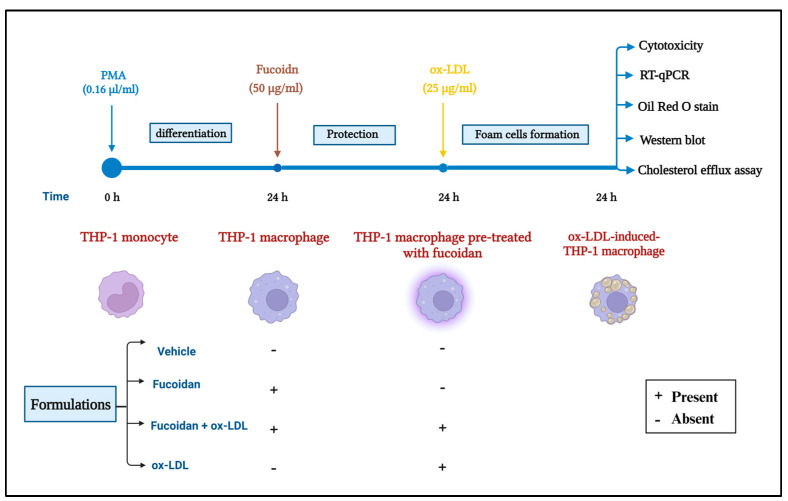
Schematic representation of the method used for monocyte culture and treatment (image created using BioRender).

THP-1 monocytes were differentiated into THP-1 macrophages by adding 160 nM of phorbol myristate acetate (PMA; Thermo Fisher Scientific, Hennigsdorf, Germany, J63916) for 24 h. THP-1 macrophages were treated with 50 µg/mL of fucoidan (≥95% HPLC, Sigma-Aldrich, St. Louis, MO, USA, F8190) for 24 h, which was prepared by dissolving 10 mg of fucoidan in 1 mL of pure distilled water. THP-1 macrophages were then transformed into foam cells for 24 h in the presence or absence of fucoidan using 25 µg/mL of ox-LDL (2.5 mg/mL, Thermo Fisher Scientific, Germany, L34357). Vehicle and fucoidan were employed as controls to compare the impact of ox-LDL induction (Figure 1).

### 2.5. Cell Viability and Proliferation Assay

The lactate dehydrogenase (LDH) test was performed as an indicator of cell membrane integrity by evaluating cell viability, following the manufacturer’s directions (Thermo Fisher Scientific, USA; 88953). THP-1 macrophages were seeded at 1 × 10^5^ cells/cm^2^ in 96-well plates for 24 h, and they were treated with fucoidan and induced with ox-LDL as mentioned above. A 50 µL amount of assay buffer was mixed with the supernatants of treated cells after they were transferred to a new plate, then incubated at room temperature (RT) for 30 min. The reaction was stopped with 50 μL of stop solution. The remaining adherent THP-1 macrophages were stained for 5 min at RT with 50 µL of crystal violet dye (crystal violet 0.2% dissolved in 10% ethanol) to evaluate the proliferation of cells. After staining, THP-1 macrophages were rinsed four times with phosphate buffer saline (PBS; Thermo Fisher Scientific, USA, 10010-015), and 50 µL of a solubilization buffer of NaH_2_PO_4_ (0.1 M) dissolved in ethanol was added to THP-1 macrophages. A BioTek Synergy HT Microplate Reader was used to measure absorption at 490 nm for LDH and 570 nm for crystal violet (Bio-Tek Instruments, Winooski, VT, USA). These experiments used independent and dependent tests, and the vitality and proliferation of the cells were expressed as a percentage related to a vehicle.

### 2.6. Quantitative Real Time PCR (qRT-PCR)

Total mRNA was isolated from THP-1 macrophages using RNeasy™ mini kit (Qiagen, Hilden, Germany, 74104), and cDNA was synthesized following the instructions of the ImProm-II Reverse Transcription Kit (Promega, Madison, WI, USA, A3800). RT-PCR was carried out using an SYBR Green BioFACTTM Kit (Daejeon, Republic of Korea, DQ383–40 h). Table 1 includes a list of all primer sequences that were employed. Expression of SR-AI, CD36 LXR-α, and ABCA1 was evaluated by a Step One PlusTM RT-PCR system (Applied Biosystems, Waltham, MA, USA). After normalization with glyceraldehyde-3-phosphate dehydrogenase (GAPDH), a housekeeping gene, relative quantification of their expression with fold change was performed using CT, 2^–ΔΔCT^, a comparative threshold method.

### 2.7. Oil Red O Staining

Lipid uptake in THP-1 macrophages was evaluated by Oil Red O stain as described by [30], with little modification. After THP-1 macrophages were seeded into 24-well plates at 5 × 10^5^ cells/mL, following treatment according to the method mentioned before, cells were washed with 300 μL of PBS three times and then fixed with 300 µL of 4% formaldehyde for 30 min at RT. After discarding the fixation solution, the cells were washed twice with water. Subsequently, cells were destained with 60% (*v*/*v*) isopropanol for 20 s, 300 μL of Oil Red O stain for 10 min, and rinsed thrice with water. The cells were counter-stained for 1 min with hematoxylin, removed, and washed with water. The stained cells were kept covered with water while being examined under an inverted microscope (INV100, BEL Engineering, Monza, Italy). The amount of the Oil Red O stain was quantified by ImageJ 1.53 K (Version 1.53 K, National Institutes of Health), which expressed the intracellular lipid content.

### 2.8. Western Blot Technique

Treated THP-1 macrophages were lysed in RIPA buffer containing a protease and phosphatase inhibitor mixture (Solarbio Life Science, Beijing, China, R00100, P1261). The concentration determination was performed using an ABCA protein assay kit (Thermo Fisher Scientific, USA, 23225). Cell lysates were mixed with an equal volume of sample buffer (Laemmli 2×, Sigma-Aldrich, USA, S3401), boiled for 5 min (*β*-actin) or incubated in ice for 10 min (ABCA1), then loaded onto 10 and 8% SDS-PAGE gel, respectively, electrophoresed at 100 V, and transferred onto PVDF membranes overnight at 15 V. The membrane was blocked after the transfer process was completed by incubation while shaking in blocking buffer [5% *w*/*v* bovine serum album (BSA), Solarbio Life Science, China, A8010), and Tween-20 in Tris-buffered saline [TBS-T, 0.1% *v*/*v*, Solarbio Life Science, China, T1080] at RT for 1 h. After incubation, the membrane was washed three times with TBS-T for 10 min each, then incubated at 4 °C with shaking with diluted primary antibody for 1 h (*β*-actin, 1/2000, Thermo Fisher Scientific, PA1-183) or overnight (ABCA1, 1/1000, Abcam, ab7360). It was further washed thrice for 10 min each with TBS-T, then the membrane was immersed in goat anti-rabbit IgG-HRP secondary antibodies (1/2000, Abcam, Cambridge, UK, ab205718) that were diluted in TBS-T for 1 h at 4 °C with constant shaking and were washed with TBS-T for a further three times for 10 min each. For the detection of membrane-bound antibodies, staining was performed with ECL Western blotting substrate (Solarbio Life Science, China, PE0010) according to manufacturer instructions, and detected by the C-DiGit Blot Scanner (LI-COR Biosciences, Lincoln, NE, USA).

### 2.9. Cholesterol Efflux Assay

A cholesterol efflux assay kit (Abcam, UK, ab196985) was utilized to quantitate the rate of cholesterol efflux using fluorescently labeled cholesterol. Briefly, THP-1 macrophages were seeded in 1 × 10^5^ cells/mL in 96-well black plates with a clear flat bottom and treated with fucoidan as described in Section 2.1. THP-1 macrophages were labeled with 100 µL of cholesterol for 16 h and then incubated in the dark at 37 °C. After labeling, the cells were rinsed with equilibrated RPMI-1640-free FBS. For cholesterol efflux to ApoA1, cells were treated with 25 µg/mL of Apo A for an extra 6 h at 37 °C. At the end of incubation time, supernatant was transferred to a new 96-well black plate, and the remaining cells were solubilized by adding 100 μL of the cell lysis buffer and shaking for 30 min at RT. Using a microplate reader (Bio-Tek Instruments, USA), plates’ fluorescence (Ex/Em = 485/530 nm) was monitored. The amount of fluorescence efflux in the supernatant divided by the sum of efflux cell lysate and supernatant was used to calculate percentage of efflux.

### 2.10. Statistical Analysis

Statistical analysis was carried out using GraphPad Prism version 9, using a one-way ANOVA followed by a Tukey’s multiple comparison test or an unpaired *t*-test. Data were described as mean ± SEM ($\bar{X}$ ± SEM), as each experiment was performed either in triplicate or two independent tests. A statistically significant difference was considered when the *p*-values were ns = non-significant, * *p* < 0.05, ** *p* < 0.001, *** *p* < 0.0005, and **** *p* < 0.0001.

## 3. Results

### 3.1. Identification of Target Proteins

**Figure 2:**
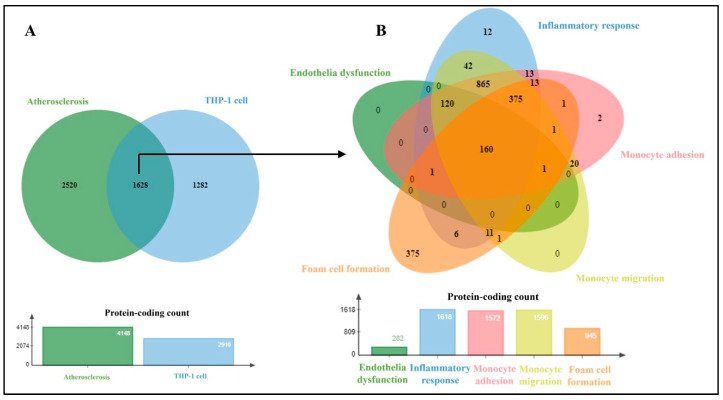
Proteins implicated in atherosclerosis and its model (THP-1 cells) using the GeneCard database. Venn diagram of overlapped target proteins in atherosclerosis and THP-1 cells (**A**), and common proteins (**B**) were classified based on the early stages of atherosclerosis.

An overview of protein-coding implicated in atherosclerosis and the study’s model are displayed in Figure 2. A total of 1628 proteins were found to have been integrated between them, and 160 proteins contributed to various phases of atherosclerosis (Supplementary Table S1). As fucoidan cannot pass through the cell membrane while interacting with receptors [31], the three major receptors involved in lipid uptake and efflux (SA-A1 (MSR1) CD36, and ABCA1) were chosen. Furthermore, the liver X receptor alpha (LXR-α, also known as nuclear receptor subfamily 1 group H member 3 (NR1H3)) induces ABCA1 expression [32].

### 3.2. Molecular Docking Analyses

**Figure 3:**
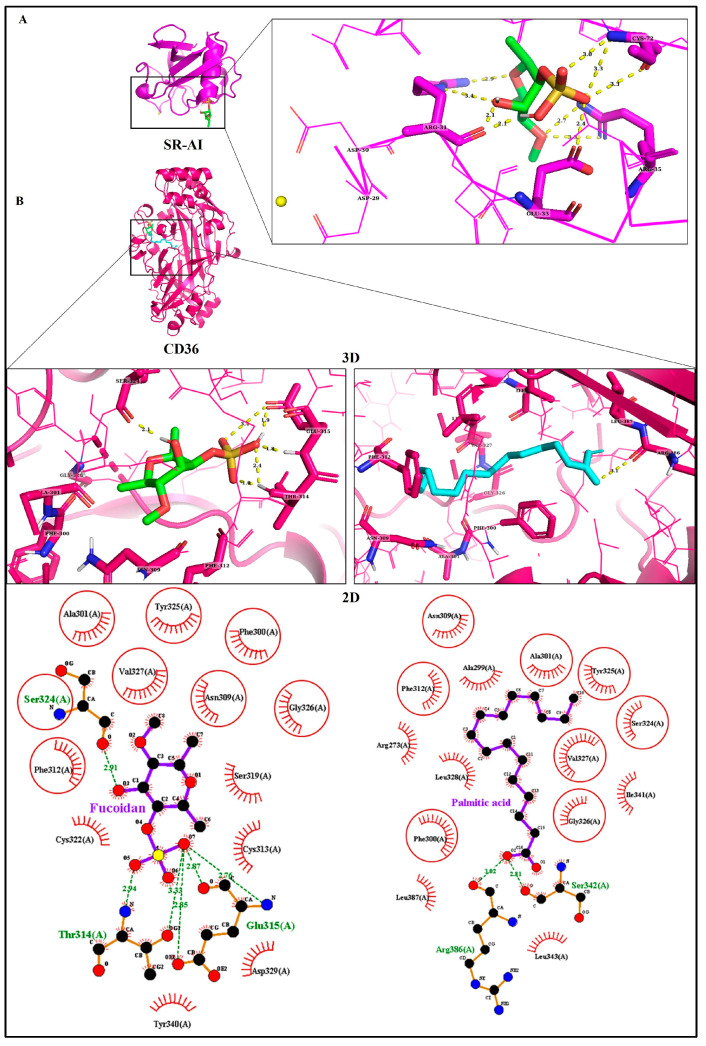
Molecular docking analysis of protein-bound ligands. (**A**) 3D structure of SR-AI bound with fucoidan and magnified ligand-binding sites. The yellow dotted lines represent the H-bonds; the yellow ball represents Ca^2+^. (**B**) 3D structure of CD36 with fucoidan and palmitic acid, colored green and cyan, respectively, with magnified ligand-binding sites and LigPlot exhibiting 2D ligand-binding interactions. The yellow and green dotted lines represent the H-bonds, the brick red represents hydrophobic interaction, and the circles represent the common residues shared by both ligands.

**Figure 4:**
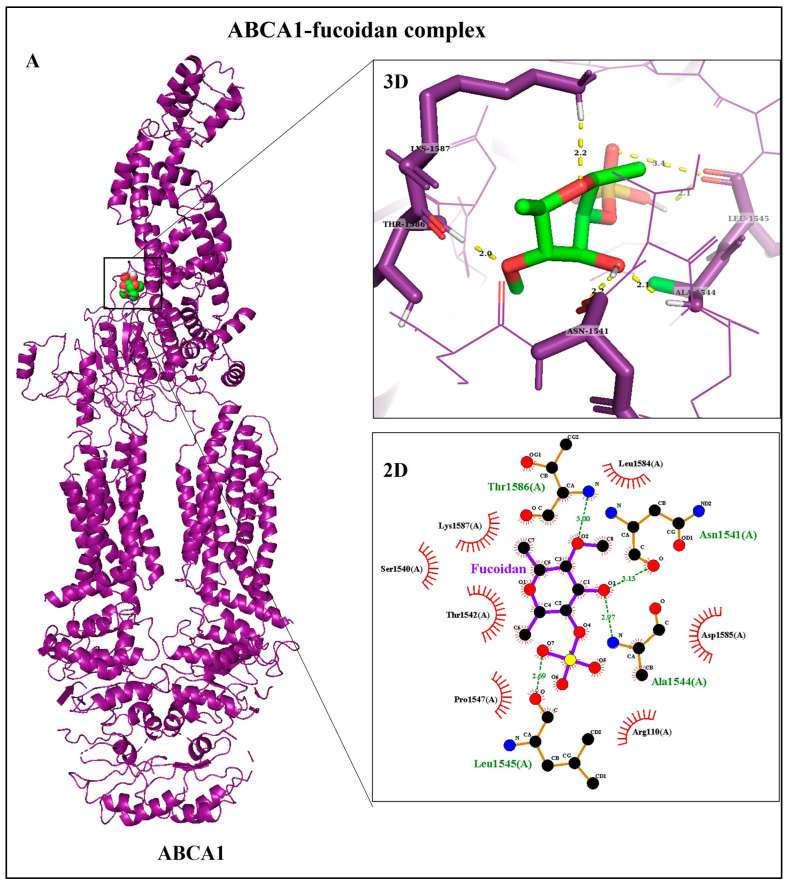

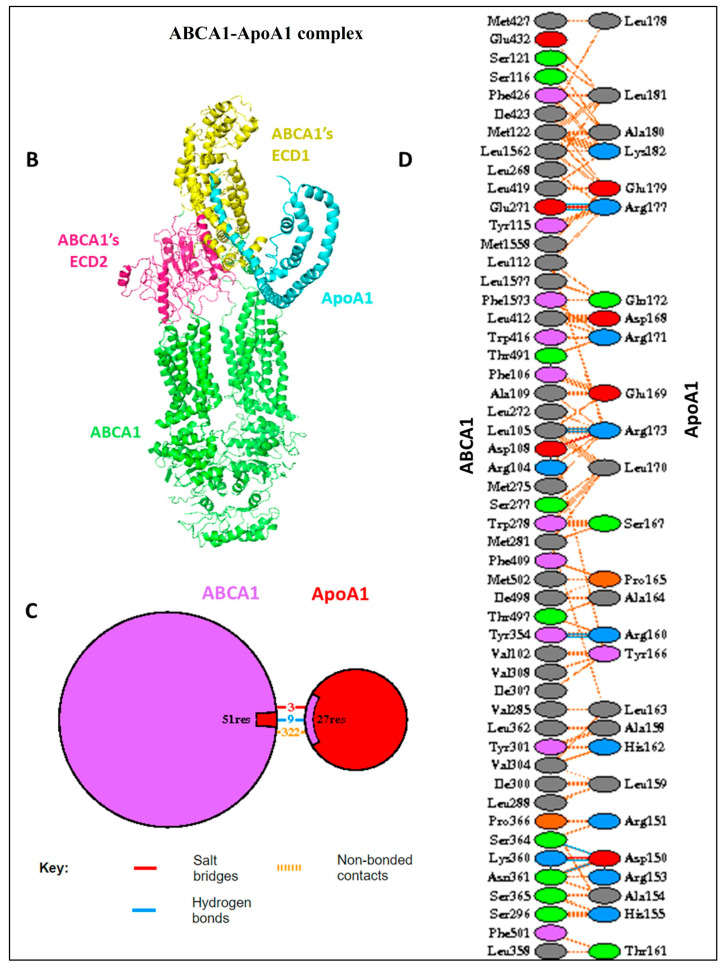
Molecular docking analysis of ABCA1. (**A**) Bound with fucoidan magnified in two dimensions; ribbon structures as 3D and LigPlot showing 2D ligand-binding sites. The yellow and green dotted lines represent the H-bonds and brick red represents hydrophobic interaction. (**B**) Best-generated model of ABCA1 bound with ApoA1 complex. (**C**,**D**) PDBsum’s interaction schematic diagram of two protein chains; the area of each circle is related to the surface area of the corresponding protein chain. Colored lines represent interacting chains between two chains, and each color refers to a different type of interaction, as displayed in the key above.

Virtual screening is the most important application of molecular docking. Molecular docking was conducted to predict binding affinities of ligands to receptors implicated in uptake and efflux of lipids. Their affinity, interactions, and occupancy (SASA) in the binding sites are depicted in Table 2. The residue binding site of SR-AI showed interaction with the fucoidan, with predicted binding energy of −7.84 kcal/mol. It forms eleven H-bonds through four residues: Arg31, Glu33, Arg35, and Cys72, displayed in Figure 3A. Unlike SR-AI, fucoidan was enclosed within the binding site of CD36, having binding energy of −11.37 kcal/mol and better occupancy. In addition, most H-bond acceptors were formed between the sulfate group and the polar amino acids Thr314 (1.9 and 2.4 Å), Glu315 (1.8, 1.9, and 3.4 Å), and Ser324 (2.1 Å) (Figure 3B). Interestingly, stronger binding of CD36 to fucoidan was noted when compared with its native ligand, palmitic acid (PLM). The interaction of CD36 with a carboxyl group of PLM and a sulfate group of fucoidan also exhibits polar interaction. While hydrophobic residues contributed to hydrophobic interactions, some of these residues were common between both ligands as Phe300, Ala301, Asn309, Phe312, Tyr325, Gly326, and Val327. On the other hand, fucoidan was docked into the ABCA1 to predict its binding affinity and stability, wherein it formed five strong H-bonds: Lys1587 (2.2 Å), Thr1586 (2.0 Å), Asn1541 (2.2 Å), Leu1545 (2.1 Å), Ala1544 (2.1 Å), and several hydrophobic interactions, mostly with residues within 1540–1587, resulting in free binding energy of −9.75 kcal/mol (Figure 4A). Figure 4B shows an overview analysis of the docked protein ABCA1–ApoA1 complexes, the best-ranked docked model of the complex representing ApoA1 bound to ABCA1’s extracellular domain 1 (ECD1), with binding energy of −427.11 kcal/mol and −4001 kcal/mol, as calculated by the ClusPro and HDOCK webservers, respectively (Table 2B). Figure 4C of PDBsum’s analysis plot illustrates how ApoA1 and ABCA1 interacted via nine H-bonds and a 322 hydrophobic interaction through residues as represented in Figure 4D.

### 3.3. The Effect of Fucoidan on Cell Viability and Proliferation of THP-1 Macrophages

**Figure 5:**
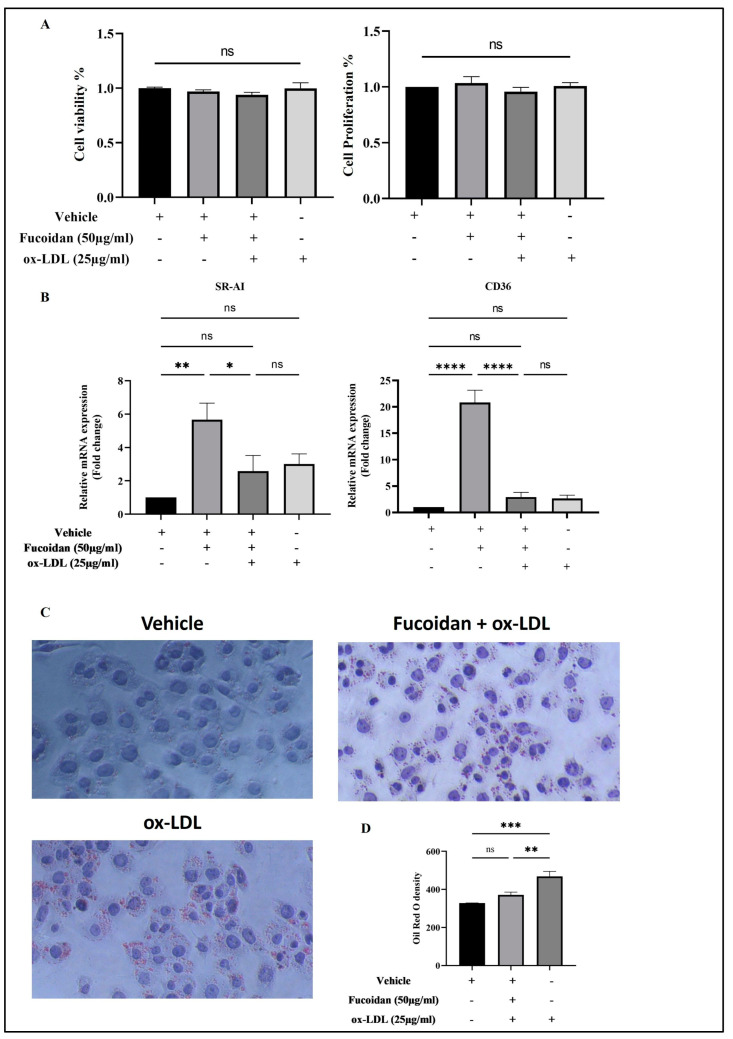
Effect of fucoidan on ox-LDL-treated THP-macrophages. These cells were treated for 24 h with vehicle and fucoidan and then they were stimulated by ox-LDL for a further 24 h. (**A**) Cell viability and proliferation of THP-1 macrophages. (**B**) Expression of genes involved in lipid uptake; RT-qPCR was used to assess the expression levels of mRNA SR-AI and CD36 in cells. (**C**) Lipid accumulation in these cells, followed by staining with Oil Red O, and visualized under a microscope; the staining density was quantified using ImageJ 1.53K (**D**). Data presented as mean ± standard error of the mean (SEM) for three independent experiments (**A**,**C**) and two independent experiments (**B**). One-way ANOVA was used for statistical analysis and Tukey’s multiple comparisons were then applied, where the *p*-values were non-significant (ns), * *p* < 0.05, ** *p* < 0.001, *** *p* < 0.0005, and **** *p* < 0.0001. (+) present and (-) absent.

An LDH assay was used to analyze the effects of fucoidan on cell viability, and the outcomes were confirmed by measuring cell proliferation using crystal violet dye. As shown in Figure 5A, 25 µg/mL of ox-LD is not significantly cytotoxic to THP-1 macrophages pre-treated with 50 µg/mL of fucoidan.

### 3.4. Fucoidan Modulates mRNA Expression of Genes Involved in ox-LDL Uptake

The influence of fucoidan on ox-LDL-induced-THP-1 macrophages through the transcription of SR-AI and CD36 is displayed in Figure 5B. In the cells pre-treated for 24 h with fucoidan, SR-AI and CD36 mRNA levels were found to be significantly higher compared to the untreated (vehicle), while their levels were considerably lower in cells incubated with 25 µg/mL of ox-LDL. It is worth mentioning that THP-1 macrophages were induced with ox-LDL only and did not express enough SR-AI or CD36.

### 3.5. Fucoidan Reduces Lipid Accumulation in THP-1 Macrophages

The impact of fucoidan on THP-1 macrophage-derived foam cells is illustrated in Figure 5C. Ox-LDL-induced cells formed significant foaming cells. In contrast, THP-1 macrophages that were pre-treated with 50 µg/mL of fucoidan showed significantly reduced intracellular lipids by two-fold in comparison to the positive control (Figure 5D).

### 3.6. Upregulation of LXR-α and ABCA1 Expression by Fucoidan

**Figure 6:**
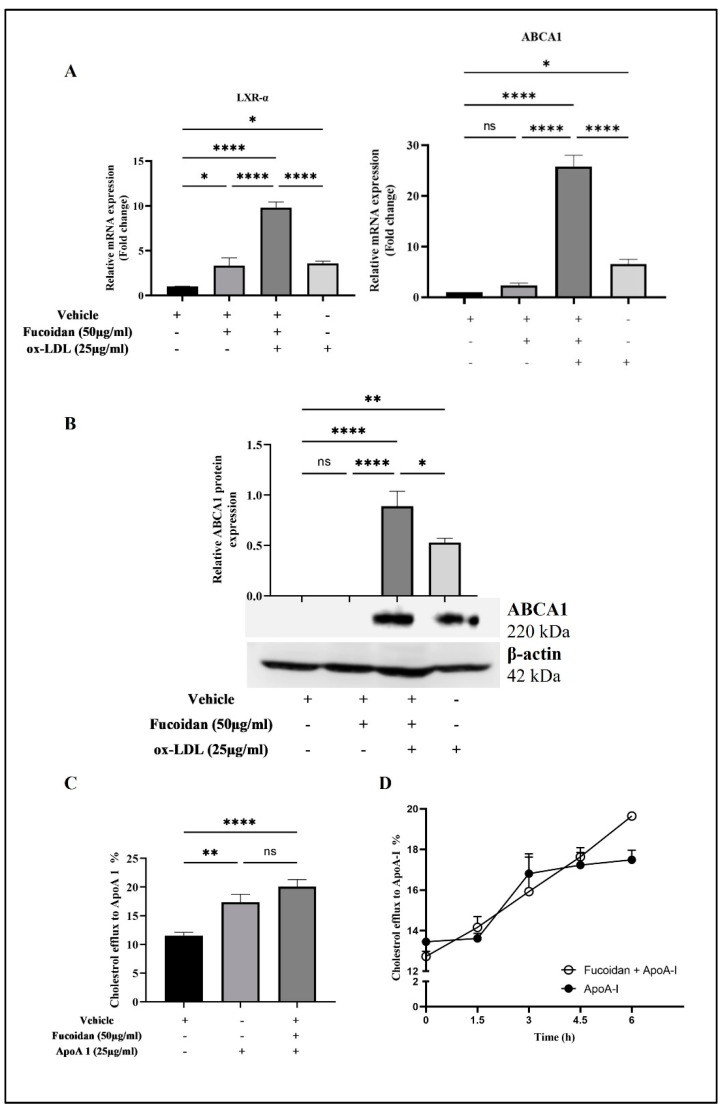
Expression level of ABCA1 in THP-1 macrophages and its ability to efflux cholesterol. Macrophages were treated with vehicle and fucoidan for 24 h and then stimulated by ox-LDL in the absence of fucoidan for a further 24 h. (**A**) Expression level of *LXR-α* and *ABCA1* mRNA in cells was determined by RT-qPCR. (**B**) Expression level of ABCA1 protein in cells was determined by Western blot; intensity of bands was analyzed using ImageJ. (**C**) Efflux of cholesterol from cells was evaluated through a high-throughput screening assay. Cells were labeled with cholesterol for 16 h after treatment, followed by stimulation with 25 μg/mL of Apo A1 in the presence and absence of fucoidan; efflux profile rate of these cells was time-dependent. (**D**) Data were described as mean ± SEM of triplicates in two (**A**,**B**,**D**) and three (**C**) independent experiments. Statistical analysis was performed by applying a one-way ANOVA (**A**–**C**) followed by Tukey’s multiple comparison test and an unpaired *t*-test (**D**), where the *p*-values were non-significant (ns), * *p* < 0.05, ** *p* < 0.001, and **** *p* < 0.0001. (+) present and (-) absent.

Fucoidan’s ability to regulate *LXR-α* and *ABCA1* expression at the mRNA level in THP-1 macrophage-derived foam cells pre-treated with 50 µg/mL fucoidan was found to be significantly upregulated by 6.1-fold and 19-fold, respectively, in cells induced by ox-LDL (Figure 6A). In addition, the results showed that there was no significant difference in ABCA1 expression in cells treated with vehicle and fucoidan, while it was significant in the case of *LXR-α*. Western blot analysis demonstrated that ABCA1 expression increases in THP-1 macrophages pre-treated with fucoidan and then induced with ox-LDL more than in cells that were unprotected (Figure 6B). Furthermore, there was no expression of ABCA1 in THP-1 macrophages that were exposed to fucoidan or a vehicle.

### 3.7. Fucoidan Regulates Cholesterol Efflux from THP-1 Macrophages

After 6 h of ApoA1 stimulation, THP-1 macrophages pre-treated with fucoidan significantly enhanced the efflux of intracellular cholesterol compared to untreated cells (negative control) and slightly increased, though not significantly, compared to cells stimulated with ApoA1 without treatment with fucoidan by 3%, as displayed in Figure 6C. It is important to note that fucoidan was observed to have gradually sustained the efflux of cholesterol and achieved the maximum efflux at 6 h, whereas the ability of foam cells to efflux cholesterol in the presence of ApoA1 was marked by a rapid rise in efflux capacity that became nearly stable in the last three hours (Figure 6D).

## 4. Discussion

**Figure 7:**
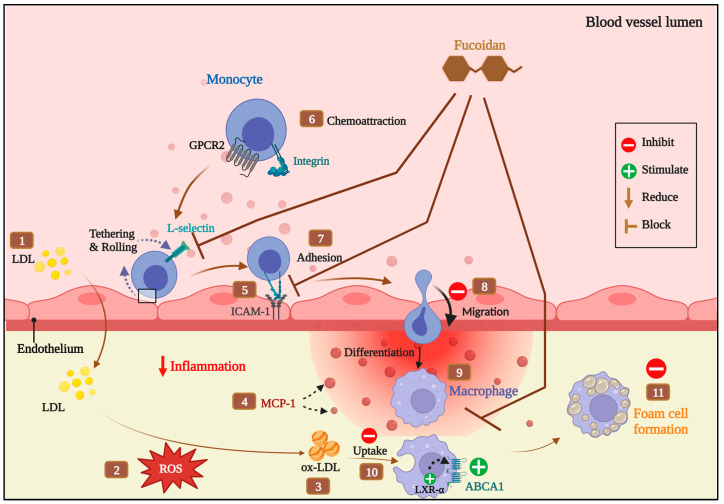
Atheroprotective mechanism of fucoidan on THP-1 cells.

Atherosclerosis is predominantly caused by a bidirectional interaction between lipids and inflammation. One of the promising sources of therapeutic approaches for atherosclerosis is targeting foam cell formation, which is affected by cholesterol homeostasis in terms of lipid uptake and cholesterol efflux [33]. Findings presented here show that fucoidan from *Fucus vesiculosus* has atheroprotective effects in ox-LDL-treated THP-macrophages; we previously explained its anti-inflammatory activities utilizing in silico and in vitro approaches [12]. This study also shows that fucoidan promotes expression of LXR-α and ABCA1 and decreases the accumulation of ox-LDL, as depicted in Figure 7. Interestingly, we report for the first time the role of fucoidan in regulating ABCA1 in THP-1 macrophages, which could be an effective treatment route for atherosclerosis.

Targeting functionally significant regions of SR reduces their binding to ox-LDL. As previously mentioned, SR-AI receptors bind a variety of negatively charged compounds, such as dextran sulfate, and many endogenous and exogenous ligands [34]. The x-ray structure of SR-AI was recently elucidated by Cheng et al., and the Ca^2+^-binding sites on the SRCR domain associated with the binding of ox-LDL were determined, which indicates that ligand recognition with Ca^2+^-binding sites is required to inhibit ox-LDL [16]. As shown by molecular docking, fucoidan has an affinity for binding to SR-AI in the binding site, even though Ca^2+^ does not recognize it, suggesting that it is possible that the fucoidan binding does not directly compete with the Ca^2+^-binding site. However, it can bind and induce conformational changes or affect the local environment, which may indirectly impact the binding of ox-LDL or modulate the function of SR-AI, resulting in upregulation of its level in cells treated with fucoidan alone, as reported in RT-qPCR, which upregulates the expression of these genes. Also, THP-1 macrophages treated with 50 μg/mL fucoidan upregulates the expression of CD36. Due to the position of PLM in the cavity, most interactions with CD36 are hydrophobic. The strong interaction of the sulfate group with CD36 might control the interaction of fucoidan in the receptor cavity and result in higher binding energy. Interestingly, punicalagin, a bioactive phenolic compound, had a higher binding affinity than PLM for CD36 [35].

Although the evidence indicates that SR-AI and CD36 efficiently limit the uptake of ox-LDL for the prevention of foam formation [36], in this study, expression of SR-AI and CD36 in THP-1 macrophages induced with 25 µg/mL ox-LDL for 24 h was not statistically significant. One explanation for these non-significant levels might be that 25 μg/mL of ox-LDL is not sufficient to induce SR expression. Moreover, studies examining bioactive substances indicated that they considerably inhibited SR-AI or CD36 expression, even though they didn’t use modified LDL as a positive control to verify their findings [37,38]. On the other hand, quercetin and myricetin have been shown in RT-qPCR and microarray-based studies to have anti-atherogenic properties in THP-1 macrophages despite their increased expression of SR-AI and CD36 at the gene level [39,40].

Furthermore, we induced foam cells using 25 µg/mL ox-LDL on THP-1 macrophages rather than the typical 50 µg/mL commonly used by other researchers [41,42]. A lower concentration of ox-LDL that is closer to physiological levels allowed us to mimic the early stages of atherosclerosis. Interestingly, in our model, we noticed foam cells were formed when staining THP-1 macrophages with Oil Red O stain, which might be due to cell uptake of ox-LDL but not through SR, and may be attributable to macropinocytosis. Cheng et al. have demonstrated that fucoidan reduced foam cell formation in THP-1 macrophages induced with 80 µg/mL ox-LDL [43]. Takala et al. showed that pinolenic acid dramatically inhibits 50% of lipid uptake and macropinocytosis in macrophages, either THP-1 or primary cultures, similar to a report that refers to eicosapentaenoic acid and docosahexaenoic acid having anti-foam cell properties that can prevent the progress of macrophage foam cells via micropinocytosis [44], despite the fact that eicosapentaenoic or docosahexaenoic acid did not inhibit mRNA expression of CD36 and SR-AI [37].

The resultant uptake of modified lipids activates the LXR/RXR signaling pathway [32,45]. It was interesting that our gene expression results indicated that ox-LDL-induced-THP-1 macrophages activate LXR-α and ABCA1 expression, whereas these genes were impressively upregulated in cells pre-treated with fucoidan, which also markedly increased expression of ABCA1 protein. These findings confirm that fucoidan enhances the expression of ABCA1 protein through mediating activation of LXR-α, which has a protective effect against atherosclerosis by promoting the reverse cholesterol transport pathway within THP-1 macrophages. To our knowledge, no study has evaluated the direct impact of fucoidan as a protective agent on ABCA1 expression. A recent study demonstrated that fucoidan reduced serum lipids and atherosclerotic plaques and inhibited NLR Family Pyrin Domain Containing 3 (NLRP3) inflammasome activation in ApoE-/- mice, as well as inhibiting NLRP3 inflammasome activation in THP-1 macrophages. As a result, inhibition of NLRP3 activation led to suppression of foam cells [43]. Fucoidan potentially reduces formation of foam cells by altering cholesterol flux-associated factors in macrophages. This was recently shown in (ApoE-/-) mice fed on a cholesterol-rich diet supplemented with fucoidan, wherein it exerted direct systemic effects. Remarkably lower foam cell formation, reduced dyslipidemia, and atherosclerosis were seen. Hence, fucoidan prevented oxLDL-mediated foam cell formation in macrophages and is associated with upregulation of the cholesterol efflux-associated SR-B1 expression but downregulation of the cholesterol influx-associated scavenger receptor SR-A1/2 expression [46]. Moreover, chayote’s water-soluble polysaccharide (WSP), when used in high quantities (400 g/mL), decreased foam cell formation in THP-1 macrophages exposed to cholesterol crystals [47] through increased expression of LXR-α, despite levels of lipid efflux receptors not being assessed in that study.

In order to examine the ability of fucoidan to mediate the efflux of cholesterol, a hallmark step in atherosclerosis progression, cellular cholesterol efflux was measured and monitored by a high-throughput screening assay. It was found that fucoidan at 50 µg/mL increased cellular cholesterol efflux to ApoA1, perhaps due to the ability of fucoidan to modulate cellular signaling pathways. One possible explanation for fucoidan’s effect on gradually regulating cholesterol release rates in time-dependent evaluation is its binding to ABCA1’s extracellular domain 2 (ECD2), which is the first domain through which lipids pass before ECD1 efflux. According to a previous study, a low dose of polysaccharides isolated from mushrooms enhances cholesterol efflux through peroxisome proliferation-activated receptor gamma/ABCA1/ATP-binding cassette transporter G1 (PPAR-γ/ABCA1/ABCG1) signaling pathways, while a high concentration of *Phellinus linteus* polysaccharide extracts (PLPEs) (100 μg/mL) reduces cholesterol efflux due to mitochondrial dysfunction [48]. One of the most prevalent post-translational modifications of proteins is glycosylation, which occurs through *O*-linked glycosylation at serine (Ser) or threonine (Thr) residues and *N*-linked glycosylation at asparagine (Asn) residues at the Asn-X-Thr/Ser recognition sequence [49,50]. Qian et al. indicated that twelve sugar moieties were incorporated into seven glycosylation sites on the ECDs, most of them in ECD2, implying their affinity for fucoidan [18]. In our docking results, fucoidan was found to be attached to ABCA1 ECD2 at Ser1540, Thr1542, and Thr1586, which suggests that it can form *O*-linked glycosylation, increasing ABCA1 stability and leading to regulated cholesterol efflux. Previous studies assessed cholesterol efflux in the presence or absence of extracellular acceptors following treatment with bioactive substances in the THP-1 macrophage model [26,51,52] without taking into account the kinetics of cholesterol efflux. Therefore, further studies are required to evaluate the kinetics of cholesterol efflux in a time-dependent manner. THP-1 macrophages activated by ox-LDL have increased ApoA1 and HDL-mediated cholesterol efflux in a dose- and time-dependent manner [53], despite the fact that no interpretation of its impact on structure or function was made.

Understanding the molecular docking interactions between fucoidan and anti-foam cell markers, as well as validation of their results using *ABCA1* gene expression, provides evidence of fucoidan’s atheroprotective properties, though this work lacks an evaluation of the cytokine profile in a time-dependent manner and signaling transduction evaluation. Furthermore, this work also paves the way for future in vivo investigations in animal models. Further immunoprecipitation or yeast two-hybrid assays can be employed to validate the predicted interactions.

## 5. Conclusions

This study provides a detailed molecular mechanism targeting the atheroprotective actions of fucoidan from *Fucus vesiculosus* in human THP-1 macrophages. The findings show that the molecular mechanisms of fucoidan in underlying atherosclerosis reduce lipid accumulation and promote cholesterol efflux via activation of ABCA1 through LXR-α in THP1 macrophage-derived foam cells. Interestingly, fucoidan aids in regulating the cholesterol efflux rate. Moreover, computational approaches were used to interpret the impact of fucoidan on protein–protein interactions. These findings could be used to comprehend the potential anti-atherosclerotic effects of fucoidan and its potential as a functional food ingredient. 

## Figures and Tables

**Table 1:** List of qPCR primer sequences.

Target Gene	Primer Sequence (5′-3′)	Reference
*GAPDH*	F: CTTTTGCGTCGCCAGCCGAG	[26]
	R: GCCCAATACGACCAAATCCGTTGACT	
*CD36*	F: ATT GCCCTTTACCTCGT	[27]
	R: GCC TTG GAT GGA AGA ACA AA	
*SR-AI*	F: ATTGCCCTTTACCTCGT	
	R: TCATTTCCTTTT CCC GTGAG	
*LXR-α*	F: AAGCCCTGCATGCCTACGT	[28]
	R: TGCAGACGCAGTGCAAACA	
*ABCA1*	F: AGTGGAAACAGTTAATGACCAG	[29]
	R: GCAGCTGACATGTTTGTCTTC	

**Table 2:** Molecular docking results: (A) Molecular docking results of interaction with anti-foam cell markers. (B) Top-ranked docking of ABCA1–ApoA1 protein complex.

A			(Protein–Ligand Docking)			
**Receptor** **(PDB ID)**	**Ligand**	**ΔG (Kcal/mol)**	**Ki**	**Type of Interactions**		**ΔSASA** **(Å^2^)**
				**H-Bonds**	**Hydrophobic**	
SR-AI (7DPX)	Fucoidan	−7.84	1.80 µM	Arg31 (2.1 Å), Glu33 (2.4 Å), Arg35 (2.2 Å), Cys72 (3.0 Å)	-	36.02
CD36 (5LGD)	Fucoidan	−11.37	4.66 nM	Thr314 (1.9 Å), Glu315 (1.8 Å), Ser324 (2.1 Å)	Phe300, Ala301, Asn309, Phe312, Cys313, Ser319, Cys322, Tyr325, Gly326, Val327, Asp329, Tyr340	138.6
	Palmitic acid	−8.30	821.7 nM	Arg386 (3.1 Å)	Ala299, Arg273, Phe300, Ala301, Asn309, Phe312, Ser324, Tyr325, Gly326, Val327, Leu328, Ile341, Leu343, Leu387	207.8
ABCA1 (5XJY)	Fucoidan	−9.75	71.68 nM	Asn1541 (2.2 Å), Ala1544 (2.1 Å), Leu1545 (2.1 Å), Thr1586 (2.0 Å), Lys1587(2.2 Å)	Arg110, Ser1540, Thr1542, Pro1547, Leu1584, Asp1585, Lys1587	142
** B **			**(Protein–protein docking)**			
**Webserver**	**Parameters**				**ABCA1–ApoA1 complex**	
ClusPro server	Lowest energy		Cluster		28	
			embers		172	
			Balanced		−4001	
HDOCK server	Summary of Rank 1		Docking score		−427.11	
			Confidence score		0.9961	
			Ligand RMSD (Å)		0.93	
PDBsum	Interface statistics		No. of salt bridges		3	
			No. of hydrogen bonds		9	
			No. of non-bonded contacts		322	

ΔG: binding free energy, Ki: inhibition constant, SASA: solvent-accessible surface area.

## Data Availability

Not applicable.

## Supplementary Materials

The following supporting information can be downloaded at: https://www.mdpi.com/article/10.3390/biomedicines11112929/s1, Table S1: List of overlapping proteins in the early stages of atherosclerosis.

## Abbreviations

ABCA1: ATP-binding cassette transporter A1; ApoA1: Apolipoprotein A; CD36: Cluster of differentiation 36; ECD2: extracellular domain 2; LDH: lactate dehydrogenase; LXR-α: liver X receptor-α; Ox-LDL: oxidized low-density lipoprotein; SASA: Solvent-accessible surface area; SR-AI: Scavenger receptor class AI.

## References

[1] Flynn M.C., Pernes G., Lee M.K.S., Nagareddy P.R., Murphy A.J. (2019). Monocytes, Macrophages, and Metabolic Disease in Atherosclerosis. Front. Pharmacol..

[2] Aldons J.L. (2000). Atherosclerosis. Nature.

[3] Lim J.P., Gleeson P.A. (2011). Macropinocytosis: An Endocytic Pathway for Internalising Large Gulps. Immunol. Cell Biol..

[4] Lin X.P., Mintern J.D., Gleeson P.A. (2020). Macropinocytosis in Different Cell Types: Similarities and Differences. Membranes.

[5] Chistiakov D.A., Bobryshev Y.V., Orekhov A.N. (2016). Macrophage-mediated Cholesterol Handling in Atherosclerosis. J. Cell. Mol. Med..

[6] Lorenzi I., von Eckardstein A., Cavelier C., Radosavljevic S., Rohrer L. (2008). Apolipoprotein A-I but Not High-Density Lipoproteins Are Internalised by RAW Macrophages: Roles of ATP-Binding Cassette Transporter A1 and Scavenger Receptor BI. J. Mol. Med..

[7] He P., Gelissen I.C., Ammit A.J. (2020). Regulation of ATP Binding Cassette Transporter A1 (ABCA1) Expression: Cholesterol-Dependent and—Independent Signaling Pathways with Relevance to Inflammatory Lung Disease. Respir. Res..

[8] Gelissen I.C., Harris M., Rye K.-A., Quinn C., Brown A.J., Kockx M., Cartland S., Packianathan M., Kritharides L., Jessup W. (2006). ABCA1 and ABCG1 Synergize to Mediate Cholesterol Export to ApoA-I. Arter. Thromb. Vasc. Biol..

[9] Shapiro M.D., Fazio S. (2016). From Lipids to Inflammation: New Approaches to Reducing Atherosclerotic Risk. Circ. Res..

[10] Toth P.P., Patti A.M., Giglio R.V., Nikolic D., Castellino G., Rizzo M., Banach M. (2018). Management of Statin Intolerance in 2018: Still More Questions Than Answers. Am. J. Cardiovasc. Drugs.

[11] Patil N.P., Le V., Sligar A.D., Mei L., Chavarria D., Yang E.Y., Baker A.B. (2018). Algal Polysaccharides as Therapeutic Agents for Atherosclerosis. Front. Cardiovasc. Med..

[12] Huwait E., Al-Saedi D.A., Mirza Z. (2022). Anti-Inflammatory Potential of Fucoidan for Atherosclerosis: In Silico and in Vitro Studies in THP-1 Cells. Molecules.

[13] Zayed A., El-Aasr M., Ibrahim A.-R.S., Ulber R. (2020). Fucoidan Characterization: Determination of Purity and Physicochemical and Chemical Properties. Mar. Drugs.

[14] Barshir R., Fishilevich S., Iny-Stein T., Zelig O., Mazor Y., Guan-Golan Y., Safran M., Lancet D. (2021). GeneCaRNA: A Comprehensive Gene-Centric Database of Human Non-Coding RNAs in the geneCards Suite. J. Mol. Biol..

[15] Bardou P., Mariette J., Escudié F., Djemiel C., Klopp C. (2014). Jvenn: An Interactive Venn Diagram Viewer. BMC Bioinform..

[16] Cheng C., Zheng E., Yu B., Zhang Z., Wang Y., Liu Y., He Y. (2021). Recognition of Lipoproteins by Scavenger Receptor Class A Members. J. Biol. Chem..

[17] Hsieh F.-L., Turner L., Bolla J.R., Robinson C.V., Lavstsen T., Higgins M.K. (2016). The Structural Basis for CD36 Binding by the Malaria Parasite. Nat. Commun..

[18] Qian H., Zhao X., Cao P., Lei J., Yan N., Gong X. (2017). Structure of the Human Lipid Exporter ABCA1. Cell.

[19] Morris G.M., Huey R., Lindstrom W., Sanner M.F., Belew R.K., Goodsell D.S., Olson A.J. (2009). AutoDock4 and AutoDockTools4: Automated Docking with Selective Receptor Flexibility. J. Comput. Chem..

[20] Ramirez D., Caballero J. (2018). Is It Reliable to Take the Molecular Docking Top Scoring Position as the Best Solution without Considering Available Structural Data?. Molecules.

[21] Mei X., Atkinson D. (2011). Crystal Structure of C-Terminal Truncated Apolipoprotein A-I Reveals the Assembly of High Density Lipoprotein (HDL) by Dimerization. J. Biol. Chem..

[22] Desta I.T., Porter K.A., Xia B., Kozakov D., Vajda S. (2020). Performance and Its Limits in Rigid Body Protein-Protein Docking. Structure.

[23] Kozakov D., Hall D.R., Xia B., Porter K.A., Padhorny D., Yueh C., Beglov D., Vajda S. (2017). The ClusPro Web Server for Protein-Protein Docking. Nat. Protoc..

[24] Yan Y., Tao H., He J., Huang S.-Y. (2020). The HDOCK Server for Integrated Protein–Protein Docking. Nat. Protoc..

[25] Laskowski R.A. (2001). PDBsum: Summaries and Analyses of PDB Structures. Nucleic Acids Res..

[26] Moss J.W.E., Davies T.S., Garaiova I., Plummer S.F., Michael D.R., Ramji D.P. (2016). A Unique Combination of Nutritionally Active Ingredients Can Prevent Several Key Processes Associated with Atherosclerosis in Vitro. PLoS ONE.

[27] Xu W., Yu L., Zhou W., Luo M. (2006). Resistin Increases Lipid Accumulation and CD36 Expression in Human Macrophages. Biochem. Biophys. Res. Commun..

[28] Saenz J., Santa-María C., Reyes-Quiroz M.E., Geniz I., Jiménez J., Sobrino F., Alba G. (2018). Grapefruit Flavonoid Naringenin Regulates the Expression of LXRα in THP-1 Macrophages by Modulating AMP-Activated Protein Kinase. Mol. Pharm..

[29] O’Morain V.L., Chan Y., Williams J.O., Alotibi R., Alahmadi A., Rodrigues N.P., Plummer S.F., Hughes T.R., Michael D.R., Ramji D.P. (2021). The Lab4P Consortium of Probiotics Attenuates Atherosclerosis in LDL Receptor Deficient Mice Fed a High Fat Diet and Causes Plaque Stabilization by Inhibiting Inflammation and Several Pro-Atherogenic Processes. Mol. Nutr. Food Res..

[30] Aldridge A., Kouroupis D., Churchman S., English A., Ingham E., Jones E. (2013). Assay Validation for the Assessment of Adipogenesis of Multipotential Stromal Cells—A Direct Comparison of Four Different Methods. Cytotherapy.

[31] Lin Z., Tan X., Zhang Y., Li F., Luo P., Liu H. (2020). Molecular Targets and Related Biologic Activities of Fucoidan: A Review. Mar. Drugs.

[32] Chinetti G., Lestavel S., Bocher V., Remaley A.T., Neve B., Torra I.P., Teissier E., Minnich A., Jaye M., Duverger N. (2001). PPAR-α and PPAR-γ Activators Induce Cholesterol Removal from Human Macrophage Foam Cells through Stimulation of the ABCA1 Pathway. Nat. Med..

[33] Wang D., Yang Y., Lei Y., Tzvetkov N.T., Liu X., Yeung A.W.K., Xu S., Atanasov A.G., Wai A., Yeung K. (2019). Targeting Foam Cell Formation in Atherosclerosis: Therapeutic Potential of Natural Products. Pharmacol. Rev..

[34] Pluddemann A., Neyen C., Gordon S. (2007). Macrophage Scavenger Receptors and Host-Derived Ligands. Methods.

[35] Huwait E., Almowallad S., Al-Massabi R., Saddeek S., Gauthaman K., Prola A. (2022). Punicalagin Targets Atherosclerosis: Gene Expression Profiling of THP-1 Macrophages Treated with Punicalagin and Molecular Docking. Curr. Issues Mol. Biol..

[36] Buckley M.L., Ramji D.P. (2015). The Influence of Dysfunctional Signaling and Lipid Homeostasis in Mediating the Inflammatory Responses during Atherosclerosis. Biochim. Et Biophys. Acta (BBA)-Mol. Basis Dis..

[37] McLaren J.E., Michael D.R., Guschina I.A., Harwood J.L., Ramji D.P. (2011). Eicosapentaenoic Acid and Docosahexaenoic Acid Regulate Modified LDL Uptake and Macropinocytosis in Human Macrophages. Lipids.

[38] Gallagher H., Williams J.O., Ferekidis N., Ismail A., Chan Y.-H., Michael D.R., Guschina I.A., Tyrrell V.J., O’Donnell V.B., Harwood J.L. (2019). Dihomo-γ-Linolenic Acid Inhibits Several Key Cellular Processes Associated with Atherosclerosis. Biochim. Et Biophys. Acta (BBA)-Mol. Basis Dis..

[39] Huwait E.A., Saddeek S.Y., Al-Massabi R.F., Almowallad S.J., Pushparaj P.N., Kalamegam G. (2021). Antiatherogenic Effects of Quercetin in the THP-1 Macrophage Model In Vitro, with Insights into Its Signaling Mechanisms Using in Silico Analysis. Front. Pharmacol..

[40] Huwait E., Almassabi R., Almowallad S., Saddeek S., Karim S., Kalamegam G., Mirza Z. (2022). Microarray Expression Profile of Myricetin-Treated THP-1 Macrophages Exhibits Alterations in Atherosclerosis-Related Regulator Molecules and LXR/RXR Pathway. Int. J. Mol. Sci..

[41] Zhao W., Wang L., Haller V., Ritsch A. (2019). A Novel Candidate for Prevention and Treatment of Atherosclerosis: Urolithin b Decreases Lipid Plaque Deposition in apoE−/− Mice and Increases Early Stages of Reverse Cholesterol Transport in Ox-LDL Treated Macrophages Cells. Mol. Nutr. Food Res..

[42] Voloshyna I., Seshadri S., Anwar K., Littlefield M.J., Belilos E., Carsons S.E., Reiss A.B. (2014). Infliximab Reverses Suppression of Cholesterol Efflux Proteins by TNF-α: A Possible Mechanism for Modulation of Atherogenesis. BioMed Res. Int..

[43] Cheng Y., Pan X., Wang J., Li X., Yang S., Yin R., Ma A., Zhu X. (2020). Fucoidan Inhibits NLRP3 Inflammasome Activation by Enhancing P62/SQSTM1-Dependent Selective Autophagy to Alleviate Atherosclerosis. Oxidative Med. Cell. Longev..

[44] Takala R., Ramji D.P., Andrews R., Zhou Y., Burston J., Choy E. (2022). Anti-Inflammatory and Immunoregulatory Effects of Pinolenic Acid in Rheumatoid Arthritis. Rheumatology.

[45] Ozasa H., Ayaori M., Iizuka M., Terao Y., Uto-Kondo H., Yakushiji E., Takiguchi S., Nakaya K., Hisada T., Uehara Y. (2011). Pioglitazone Enhances Cholesterol Efflux from Macrophages by Increasing ABCA1/ABCG1 Expressions via PPARγ/LXRα Pathway: Findings from in Vitro and Ex Vivo Studies. Atherosclerosis.

[46] Fernandes-Braga W., Aguilar E.C., Navia-Pelaez J.M., Ávila D.L., Rezende L., de Oliveira Andrade L., Miranda S.E.M., Barros A.L.B.d., Capettini L.d.S.A., Alvarez-Leite J.I. (2023). The Atheroprotective Role of Fucoidan Involves the Reduction of Foam Cell Formation by Altering Cholesterol Flux-Associated Factors in Macrophages. Biochem. Biophys. Res. Commun..

[47] Castro-Alves V.C., Shiga T.M., do Nascimento J.R.O. (2019). Polysaccharides from Chayote Enhance Lipid Efflux and Regulate NLRP3 Inflammasome Priming in Macrophage-like THP-1 Cells Exposed to Cholesterol Crystals. Int. J. Biol. Macromol..

[48] Li X., Li Y., Cheng Z., Cai X., Wang H. (2015). The Effects of Phellinus Linteus Polysaccharide Extracts on Cholesterol Efflux in Oxidized Low-Density Lipoprotein–Loaded THP-1 Macrophages. J. Investig. Med..

[49] Hansen J.E., Lund O., Engelbrecht J., Bohr H., Nielsen J.O., Hansen J.E.S., Brunak S. (1995). Prediction of O-Glycosylation of Mammalian Proteins: Specificity Patterns of UDP-GalNAc:Polypeptide N-Acetylgalactosaminyltransferase. Biochem. J..

[50] Steen P.V.d., Rudd P.M., Dwek R.A., Opdenakker G. (1998). Concepts and Principles of O-Linked Glycosylation. Crit. Rev. Biochem. Mol. Biol..

[51] Almassabi R.F., Huwait E.A., Almowallad S.J., Saddeek S.Y., Gauthaman K. (2021). In Vitro: The Modulating Effect of Myricetin on the Atherosclerosis Related Processes in THP1 Macrophages. J. Pharm. Res. Int..

[52] Tan C., Zhou L., Wen W., Xiao N. (2021). Curcumin Promotes Cholesterol Efflux by Regulating ABCA1 Expression through miR-125a-5p/SIRT6 Axis in THP-1 Macrophage to Prevent Atherosclerosis. J. Toxicol. Sci..

[53] Xu X., Li Q., Pang L., Huang G., Huang J., Shi M., Sun X., Wang Y. (2013). Arctigenin Promotes Cholesterol Efflux from THP-1 Macrophages through PPAR-γ/LXR-α Signaling Pathway. Biochem. Biophys. Res. Commun..

