# Supplementary material for: Atheroprotective Effect of Fucoidan in THP-1 Macrophages by Potential Upregulation of ABCA1
Source: Biomedicines. 2023 Oct 30;11(11):2929. doi: 10.3390/biomedicines11112929 (PMC10669811; doi:10.3390/biomedicines11112929)
Supplement: Supplementary file 1 [file biomedicines-11-02929-s001.zip › biomedicines-2674023-supplementary.pdf]

Table S1: List of overlapping proteins in the early stages of atherosclerosis.

Protein Symbol	Description
ABCA1	ATP Binding Cassette Subfamily A Member 1
LPL	Lipoprotein Lipase
PPARG	Peroxisome Proliferator Activated Receptor Gamma
LPA	Lipoprotein(A)
CCL2	C-C Motif Chemokine Ligand 2
IL6	Interleukin 6
OLR1	Oxidized Low Density Lipoprotein Receptor 1
ADIPOQ	Adiponectin, C1Q And Collagen Domain Containing
SELE	Selectin E
ACE	Angiotensin Converting Enzyme
MSR1	Macrophage Scavenger Receptor 1
TLR4	Toll Like Receptor 4
TNF	Tumor Necrosis Factor
ELN	Elastin
NOS3	Nitric Oxide Synthase 3
MMP9	Matrix Metallopeptidase 9
SELP	Selectin P
ICAM1	Intercellular Adhesion Molecule 1
VCAM1	Vascular Cell Adhesion Molecule 1
NLRP3	NLR Family Pyrin Domain Containing 3
NR1H3	Nuclear Receptor Subfamily 1 Group H Member 3
ESR1	Estrogen Receptor 1
IL1B	Interleukin 1 Beta
AGER	Advanced Glycosylation End-Product Specific Receptor
EDN1	Endothelin 1
VWF	Von Willebrand Factor
MPO	Myeloperoxidase
CD36	CD36 Molecule
VEGFA	Vascular Endothelial Growth Factor A
THBD	Thrombomodulin
IL18	Interleukin 18
TGFB2	Transforming Growth Factor Beta 2
PTGS2	Prostaglandin-Endoperoxide Synthase 2
F3	Coagulation Factor III, Tissue Factor
CXCL8	C-X-C Motif Chemokine Ligand 8
HMOX1	Heme Oxygenase 1
CD40	CD40 Molecule
TGFB3	Transforming Growth Factor Beta 3
TGFBR1	Transforming Growth Factor Beta Receptor 1
TGFB1	Transforming Growth Factor Beta 1
KNG1	Kininogen 1

NOS2	Nitric Oxide Synthase 2
TLR2	Toll Like Receptor 2
PECAM1	Platelet And Endothelial Cell Adhesion Molecule 1
CAT	Catalase
IRAK1	Interleukin 1 Receptor Associated Kinase 1
TP53	Tumor Protein P53
F5	Coagulation Factor V
F2	Coagulation Factor II, Thrombin
EDNRA	Endothelin Receptor Type A
CX3CL1	C-X3-C Motif Chemokine Receptor 1
SIRT1	Sirtuin 1
MMP1	Matrix Metallopeptidase 1
IGF1	Insulin Like Growth Factor 1
IL1RN	Interleukin 1 Receptor Antagonist
CAV1	Caveolin 1
IFNG	Interferon Gamma Receptor 1
SOD1	Superoxide Dismutase 1
PTGS1	Prostaglandin-Endoperoxide Synthase 1
CXCL12	C-X-C Motif Chemokine Ligand 12
CDKN2A	Cyclin Dependent Kinase Inhibitor 2A
TRAF6	TNF Receptor Associated Factor 6
RELA	RELA Proto-Oncogene, NF-KB Subunit
IL1A	Interleukin 1 Alpha
MAPK14	Mitogen-Activated Protein Kinase 14
NFE2L2	NFE2 Like BZIP Transcription Factor 2
HIF1A	Hypoxia Inducible Factor 1 Subunit Alpha
CCL5	C-C Motif Chemokine Ligand 5
NOX4	NADPH Oxidase 4
THBS1	Thrombospondin 1
NFKB1	Nuclear Factor Kappa B Subunit 1
SELPLG	Selectin P Ligand
PGF	Placental Growth Factor
RHOA	Ras Homolog Family Member A
MAPK1	Mitogen-Activated Protein Kinase 1
CASP3	Caspase 3
APLN	Apelin
LGALS3	Galectin 3 Binding Protein
RHOD	Ras Homolog Family Member D
CYBB	Cytochrome B-245 Beta Chain
SP1	Sp1 Transcription Factor
PLG	Plasminogen
PARP1	Poly(ADP-Ribose) Polymerase 1
MAPK3	Mitogen-Activated Protein Kinase 3
BSG	Basigin (Ok Blood Group)

PDGFB	Platelet Derived Growth Factor Subunit B
ITGB2	Integrin Subunit Beta 2
ANXA5	Annexin A5
FN1	Fibronectin 1
CTNNB1	Catenin Beta 1
CXCL10	C-X-C Motif Chemokine Ligand 10
IL33	Interleukin 33
KLF2	KLF Transcription Factor 2
APOL1	Apolipoprotein L1
NOS1	Nitric Oxide Synthase 1
BCL2	BCL2 Apoptosis Regulator
VDR	Vitamin D Receptor
EGF	Epidermal Growth Factor
CASP1	Caspase 10
CXCL1	C-X-C Motif Chemokine Ligand 1
AR	Androgen Receptor
CXCR2	C-X-C Motif Chemokine Receptor 2
CD59	CD59 Molecule (CD59 Blood Group)
FAS	Fas Cell Surface Death Receptor
ITLN1	Intelectin 1
SRC	SRC Proto-Oncogene, Non-Receptor Tyrosine Kinase
MAPK8	Mitogen-Activated Protein Kinase 8
PLAUR	Plasminogen Activator, Urokinase Receptor
ITGAL	Integrin Subunit Alpha L
S100A9	S100 Calcium Binding Protein A9
FURIN	Furin, Paired Basic Amino Acid Cleaving Enzyme
PLAU	Plasminogen Activator, Urokinase
SERPINF1	Serpin Family F Member 1
ABCB1	ATP Binding Cassette Subfamily B Member 1
BMP2	Bone Morphogenetic Protein 2
S100A8	S100 Calcium Binding Protein A8
CTSB	Cathepsin B
PDGFA	Platelet Derived Growth Factor Subunit A
HBEGF	Heparin Binding EGF Like Growth Factor
MYD88	MYD88 Innate Immune Signal Transduction Adaptor
CD44	CD44 Molecule (Indian Blood Group)
MERTK	MER Proto-Oncogene, Tyrosine Kinase
HPSE	Heparanase
PDGFRB	Platelet Derived Growth Factor Receptor Beta
ITGA4	Integrin Subunit Alpha 4
TF	Transferrin
NEU1	Neuraminidase 1
FOS	Fos Proto-Oncogene, AP-1 Transcription Factor Subunit
CDKN1B	Cyclin Dependent Kinase Inhibitor 1B

CNR2	Cannabinoid Receptor 2
BAX	BCL2 Associated X, Apoptosis Regulator
GLP1R	Glucagon Like Peptide 1 Receptor
SLC2A1	Solute Carrier Family 2 Member 1
ITGAV	Integrin Subunit Alpha V
PTAFR	Platelet Activating Factor Receptor
CD47	CD47 Molecule
P2RX7	Purinergic Receptor P2X 7
HSPG2	Heparan Sulfate Proteoglycan 2
PTK2	Protein Tyrosine Kinase 2
ADPRH	ADP-Ribosylarginine Hydrolase
VIM	Vimentin
LYN	LYN Proto-Oncogene, Src Family Tyrosine Kinase
CSK	C-Terminal Src Kinase
ANXA1	Annexin A11
MUC1	Mucin 1, Cell Surface Associated
CHGA	Chromogranin A
FGFR2	Fibroblast Growth Factor Receptor 2
CCL20	C-C Motif Chemokine Ligand 20
GSN	Gelsolin
RAB5A	RAB5A, Member RAS Oncogene Family
PLPP3	Phospholipid Phosphatase 3
BMP7	Bone Morphogenetic Protein 7
WT1	SWT1 RNA Endoribonuclease Homolog
ISG15	ISG15 Ubiquitin Like Modifier
CDH1	Cadherin 13
ATP6V0A2	ATPase H+ Transporting V0 Subunit A2
TGFA	Transforming Growth Factor Alpha
ICAM3	Intercellular Adhesion Molecule 3
TUBB	Tubulin Beta Class I
FUT4	Fucosyltransferase 4
